# Research Progress on the Functions of Gasotransmitters in Plant Responses to Abiotic Stresses

**DOI:** 10.3390/plants8120605

**Published:** 2019-12-13

**Authors:** Yandong Yao, Yan Yang, Changxia Li, Dengjing Huang, Jing Zhang, Chunlei Wang, Weifang Li, Ni Wang, Yuzheng Deng, Weibiao Liao

**Affiliations:** College of Horticulture, Gansu Agricultural University, Lanzhou 730070, China; yyd614636237@163.com (Y.Y.); yangyan1061214181@163.com (Y.Y.); licx_gsau@163.com (C.L.); huangdj3032@163.com (D.H.); ZhKara@163.com (J.Z.); wangchunlei@gsau.edu.cn (C.W.); liwf0508@163.com (W.L.); wangni19941011@163.com (N.W.); dengyz0830@163.com (Y.D.)

**Keywords:** gasotransmitters, abiotic stress, production, antioxidant enzyme, interaction

## Abstract

Abiotic stress is one of the major threats affecting plant growth and production. The harm of abiotic stresses includes the disruption of cellular redox homeostasis, reactive oxygen species (ROS) production, and oxidative stress in the plant. Plants have different mechanisms to fight stress, and these mechanisms are responsible for maintaining the required homeostasis in plants. Recently, the study of gasotransmitters in plants has attracted much attention, especially for abiotic stress. In the present review, abiotic stressors were mostly found to induce gasotransmitter production in plants. Meanwhile, these gasotransmitters can enhance the activity of several antioxidant enzymes, alleviate the harmfulness of ROS, and enhance plant tolerance under various stress conditions. In addition, we introduced the interaction of gasotransmitters in plants under abiotic stress. With their promising applications in agriculture, gasotransmitters will be adopted in the near future.

## 1. Introduction

In nature, plants are constantly challenged by a variety of abiotic stressors, such as heavy metals, low or high temperature, drought or osmotic pressure, salt, and ultraviolet irradiation. Several studies have found that plants are affected in their height, leaf morphology, and stomatal openness under abiotic stress [1,2,3]. In addition, the physiological metabolism of plants is disordered, whereby the contents of proline (Pro), electrolyte leakage (EC), malondialdehyde (MDA), and hydrogen peroxide (H_2_O_2_) are changed by abiotic stress [4,5]. At the same time, as the activities of some antioxidant enzymes change, the production of ROS is considered to be a commonplace factor in plants’ responses to abiotic stress [6,7,8]. Researchers have found that the redox environment in a cell is maintained within a “Goldilocks zone”, wherein ROS production is sufficiently counterbalanced by antioxidant capacity and quality control systems [9]. However, when faced with persistent oxidative stress, the redox environment could be pushed outside of this Goldilocks zone, where cell death and damage ensues [9]. Therefore, adjusting the plant’s redox homeostasis is a necessary aspect of abiotic stress resistance. To adapt to such stresses, plants have developed detailed mechanisms to perceive external signals and to embody adaptive responses with suitable physiological and morphological changes [10].

Gasotransmitters are small gas molecules that are generated by organisms and transmit biological signals. Research on gasotransmitters is rapidly expanding and knowledge regarding the potential of gasotransmitters in biology and medicine is accumulating [11]. Gasotransmitters, such as hydrogen gas (H_2_), hydrogen sulfide (H_2_S), nitric oxide (NO), carbon monoxide (CO), and methane (CH_4_), are unique and regulate specific biological functions. Gasotransmitters have long been of great interest in a wide range of fields. Over the past few decades, the roles of these signaling molecules have been extensively studied for their biological applications. Recently, the emissions of endogenous gasotransmitters in plants have been widely studied and analyzed, thereby providing information to facilitate our understanding of new gasotransmitter signaling pathways. Previous studies found that in response to abiotic stressors, plants usually produce these gasotransmitters [12,13,14]. In addition, there is now considerable evidence to show that gasotransmitters can play a pivotal role in enhancing plant tolerance [2,15,16]. Subsequently, biological gases from complex extra and intracellular pathways and gas mediators may regulate many processes in an antagonistic or synergistic way.

In the present review, we introduce the production of gasotransmitters in plants under abiotic stress. Meanwhile, we focused on the recent advances in the roles of gasotransmitters under abiotic stresses and their interaction with other gasotransmitters.

## 2. Production of Gasotransmitters under Adverse Conditions

### 2.1. Hydrogen Gas (H_2_)

**Figure 1:**
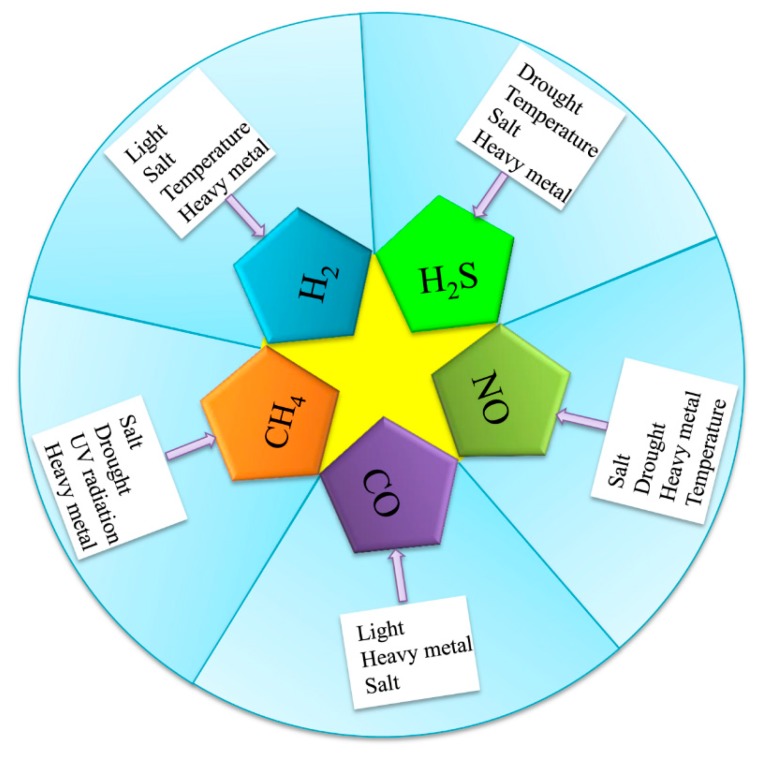
Multiple environmental stressors can induce gasotransmitter production in plants. Abiotic stressors (drought, salt, heavy metal, temperature, light, and UV radiation) induced the generation of gasotransmitters (H_2_, H_2_S, NO, CO, and CH_4_).

Early in the 20th century, the emissions of H_2_ were first observed in bacteria by Stephenson and Stickland [17], who found that H_2_ is produced due to the presence of hydrogenase in bacteria. Subsequent studies showed that H_2_ could also be produced by green algae and higher plants [18]. Renwick et al. [19] found that lettuce seed germination could release H_2_ under bright light. Recently, studies have discovered that plants can produce H_2_ under abiotic stresses. For example, H_2_ production is induced by salt stress in rice [20] (Figure 1) and alfalfa [21]. Xu et al. [22] also found that H_2_ is produced in rice under a cold stress stimulation. The exposure of alfalfa to paraquat stress increased endogenous H_2_ production [23]. Under aluminum stress, rice also produced H_2_ [16]. Meanwhile, H_2_ production was found to be induced by abscisic acid, ethylene, and jasmonate acid; salt; and drought stress in rice [24] (Figure 1). However, although some evidence of H_2_ is produced in plants under abiotic stresses, H_2_ does not have a clear mechanism of production in plants.

### 2.2. Hydrogen Sulfide (H_2_S)

H_2_S is thought to be a key signalling molecule, and there is growing interest in the roles of H_2_S in plants [25]. H_2_S is produced in response to many abiotic stressors, including drought, temperature, and heavy metal stress [26]. Several studies have demonstrated that drought stress induces H_2_S production in *Arabidopsis thaliana* [2,27] (Figure 1). Meanwhile, abscisic acid (ABA) application improved the endogenous H_2_S content in wheat under drought stress [28]. In addition, increasing evidence has indicated that temperature stress also induces the release of H_2_S in grape [29] and cucumber [30]. Cheng et al. [31] found that H_2_S generation in poplars is rapidly induced by high temperatures. Shi et al. [32] found that endogenous H_2_S is evidently induced by cadmium (Cd) stress treatment in Bermuda grass. Lead exposure also induced H_2_S production in cauliflower [33]. Valivand et al. [34] also reported that nickel (Ni) stress increased H_2_S content in zucchini. Khan et al. [35] showed that wheat seedlings released H_2_S under osmotic stress. Glyphosate-induced H_2_S released from *Arabidopsis* [36]. Interestingly, Aghdam et al. [37] found that the treatment of hawthorn fruit by exogenous H_2_S under cold stress can lead to a release of endogenous H_2_S. Therefore, Jost et al. [38] suggested that H_2_S is produced from L-cysteine in the presence of hydrogen cyanide, which is catalyzed by ß-cyanoalanine synthase in plants, though the mechanism of production under abiotic stress remains to be further studied.

### 2.3. Nitric Oxide (NO)

One of the oldest (and still popular) topics in plant NO research is the synthesis of this gaseous molecule [39]. In higher plants, NO can be generated by oxidative and reductive mechanisms involving both enzymatic and nonenzymatic systems [40]. Klepper [41] was the first to observe the production of NO in herbicide-treated soybean. Subsequently, NO was shown to be produced under salt stress in tobacco [42] and *Arabidopsis* [43] (Figure 1). The authors found that the NO increase in tobacco leaves under stress conditions was due to the induction of nitrate reductase. Thus, NO generation could be closely related to plant nitrate assimilation. Meanwhile, exogenous NO and arginine stimulated the production of endogenous NO in wheat under drought conditions [44]. Liao et al. [45] found that marigold explants also stimulated NO production under drought stress. Exogenous CH_4_ and sodium nitroprusside triggered the production of NO under osmotic stress [46]. Recently, studies have found that heavy metal aluminum stress induced NO generation in wheat [5,47] and peanut [48]. Moreover, cadmium induced NO production in *Arabidopsis* [49] and in the lichen *Ramalina farinacea* [50]. Significantly, NO production was found to be induced by phytohormone and other signaling molecules, such as indole-3-butyric acid [51], 1-methylcyclopropene [52], ABA [53], and hydrogen-rich Water (HRW) [54].

### 2.4. Carbon Monoxide (CO)

The production of CO in plants was first discovered by Wilks [55]. Subsequently, Tarr et al. [56] reported the direct emission of CO by lima beans exposed to sunlight. Moreover, abiotic stressors may induce CO production in plants. CO production was reported in the roots of *Medicgo Sative* under cadmium stress [57] (Figure 1). Zilli et al. [58] found that soybean leaves and roots release CO under salt stress. Light induced the release of CO from Arabidopsis by stimulating plant pigment B [14]. Therefore, CO was produced mainly by enhancing heme oxygenase (HO) activity.

### 2.5. Methane (CH_4_)

Firstly, Nouchi et al. [59] discovered that CH_4_ was produced in rice. Then, Keppler et al. [60] reported that rice paddies produced CH_4_ in aerobic conditions. Meanwhile, CH_4_ was found under ultraviolet radiation in tobacco [61], as well as in *Betula populifolia*, *Crataegus laevigata*, *Malus domestica*, *Plantago lanceolata*, *Quercus robur*, *Salix alba*, *Salix caprea* [62], and *Brassica oleracea* [63]. Brüggemann et al. [64] found that poplar released CH_4_ under low light conditions. Alfalfa encourages CH_4_ production under salt stress [65]. Han et al. [66] reported that polyethylene glycol increases CH_4_ production in maize. Some researchers have demonstrated that alfalfa produces CH_4_ under heavy metal stress, such as from copper (Cu) [67], aluminum (Al) [13], or cadmium (Ca) [68]. Pea leaves release CH_4_ at high temperatures [12]. Recently, Martel and Qaderi [69] found that CH_4_ is also produced in canola under blue light (Figure 1). Interestingly, Messenger et al. [70] found that CH_4_ is produced in citrus fruit under ultraviolet radiation (UV) when UV reacts with its photosensitizer to produce hydroxy radicals (·OH), which causes the pectin methyl group to form CH_4_.

## 3. The Role of Gasotransmitters under Adverse Conditions

### 3.1. Hydrogen Gas (H_2_)

#### 3.1.1. Heavy Metal Stress

Heavy metals cause serious environmental pollution across the world, threatening human health and plant growth development. Cd is a toxic metal that can be rapidly absorbed by roots and accumulated in diverse plants, thereby hampering alfalfa and cole seedling growth. Cui et al. [6] observed that hydrogen rich water pretreatment (HRW, which is a safe, economical, and easily available method that provides a valuable approach to investigate the physiological functions of H_2_ in the scientific field), could alleviate Cd-induced growth inhibition in alfalfa seedlings. The authors found that HRW attenuated Cd toxicity by enhancing antioxidant enzyme activities, including SOD, POD, APX, and enhancing the transcripts levels of relevant antioxidant genes, such as Cu, Zn-SOD, and APX1/2 in the root tissues of alfalfa plants. HRW enhanced the antioxidant capacity of Chinese cabbage under Cd stress and decreased ROS production [8] (Table 1). In addition, glutathione (GSH) is considered to be the main thiol-disulfide redox buffer of the cell. Research has demonstrated that the content of GSH reduction is important in maintaining a redox environment [71]. H_2_ might be an important regulatory factor in improving the tolerance of *Brassica campestris* seedlings against Cd, primarily by governing GSH homeostasis [72]. Dai et al. [73] suggested that HRW alleviated Cd toxicity chiefly by reducing oxidative damage promoting sulfur compound metabolism and maintaining nutrient element homeostasis in alfalfa. Al toxicity may also destroy redox homeostasis in plants. Further, 75% HRW pretreatment could significantly mitigate Al toxicity in maize seedlings, chiefly through re-establishing redox homeostasis and maintaining nutrient homeostasis [74]. Meanwhile, Xu et al. [16] observed that HRW mitigates the germination inhibition of rice seeds caused by Al stress, and HRW-regulated miRNA and its target gene expression might be an important reason for this. Additionally, exposure of alfalfa seedlings to Al not only increased NO production but also led to an inhibition of root elongation [75]. However, HRW pretreatment decreased NO production, ultimately alleviating the toxicity of Al in alfalfa seedling roots [75]. Mercury (Hg) stress could also cause oxidative damage to alfalfa, while H_2_ treatment could reduce the accumulation of Hg in alfalfa seedlings and consequently enhance plant growth upon Hg exposure [76] (Table 1). H_2_ has been indicated to relieve abiotic stress in cells, thus improving responses to stress challenges in plants [73]. There are two spin states (para- and ortho-) of H_2_ [77]. Some enzymes involved in ROS metabolism and signaling have been shown to be affected by magnetic fields. The crosstalk between the magnetic field and H_2_ was suggested to be a possible mechanism for changing cell functions [77]. Therefore, H_2_ alleviates heavy metal toxicity mainly by decreasing ROS content and enhancing the activities of typical antioxidant enzymes.

#### 3.1.2. Salt and Temperature Stresses

Normally, salinity retards seed germination and inhibits seedling growth, thus significantly reducing crop yields. Sustaining a highly efficient antioxidant system tightly regulated by different groups of transcription factors is important for plants to be able to scavenge salinity-triggered ROS overproduction. In *Arabidopsis*, H_2_ (50% HRW) pretreatment modulated the gene and protein expression of the zinc(Zn)-finger transcription factor ZAT10/12 and antioxidant defense-related enzymes, thereby significantly counteracting NaCl-induced ROS excessive production and lipid peroxidation [21] (Table 1). Moreover, H_2_ also sustained the ion homeostasis of *Arabidopsis* by regulating the antiporters [21]. Furthermore, HRW enhanced isozymatic activities and the corresponding transcripts of antioxidant enzymes, thus alleviating salt stress in rice during seed germination [20]. Meanwhile, the authors found that the ratio of potassium to sodium in both the shoot and root parts was enhanced by HRW under salt stress [20]. Therefore, H_2_ might regulate antioxidant systems, Zn-finger transcription factors, and ion homeostasis, thereby enhancing plant salt resistance.

Heat stress is a main limiting factor for plant photosynthesis and membrane stability. Recently, Chen et al. [78] found that H_2_ is involved in the mitigation of heat-induced oxidative stress by decreasing ROS accumulation, thus enhancing antioxidant enzyme activities and photosynthesis in cucumber seedlings. Additionally, HRW might protect intracellular proteins from heat-induced damage by improving the expression level of heat shock protein 70 [78]. Also, in rice seedlings, genetic evidence has shown that H_2_ might enhance cold tolerance by re-establishing redox homeostasis through regulating *miR398* and *miR319* [22] (Table 1). We deduced that the antioxidant enzyme and heat shock proteins play important roles in H_2_-induced temperature stress resistance.

#### 3.1.3. Ultraviolet Radiation

HRW can enhance UV-A-induced anthocyanin accumulation in radish sprouts and re-establish ROS homeostasis [79] (Table 1). Moreover, a molecular analyses indicated that anthocyanin biosynthesis-related genes were upregulated markedly in radish sprouts by HRW plus UV-A treatment [79]. A similar result was reported for alfalfa in a study by Xie et al. [80], which demonstrated that the biosynthesis of (iso) flavonoids can be enhanced by HRW under UV-B irradiation in alfalfa. In addition, HRW enhanced anthocyanin accumulation, total phenolic content, and alleviated oxidative damage to immature radish microgreens under short wavelength light [81]. Furthermore, HRW might be involved in enhancing key enzyme activities and upregulating of the expression of genes for anthocyanin biosynthesis [81]. These discoveries indicate that H_2_ acts as a novel cytoprotective promoter of anthocyanin accumulation, enhancing antioxidant enzyme activity and improving plant ultraviolet radiation tolerance.

#### 3.1.4. Drought and Paraquat Stresses

In response to an ABA or water deficit, HRW-pretreated alfalfa seedlings rapidly accumulated higher contents of H_2_O_2_ and showed more tolerance to drought stress [65]. Jin et al. [82] found that 50% HRW regulated stomatal closure under drought stress in alfalfa, which was dependent on ABA. The authors also found that HRW could significantly increase the apoplastic potential of hydrogen (pH) of leaves under drought stress. Thus, H_2_, as a new regulatory mechanism, may enhance alfalfa tolerance to drought stress by elevating H_2_O_2_ levels and increasing the apoplastic pH. Additionally, under paraquat-induced oxidative stress, alfalfa treated with HRW showed decreased superoxide anion radical levels and alleviated oxidative stress via heme oxygenase-1 (HO-1; a ubiquitous enzyme catalyzing degradation of heme to produce CO) signalling [23] (Table 1). Therefore, H_2_ may act as a new bioactive molecule in enhancing plant tolerance to drought and paraquat-induced oxidative stress.

### 3.2. Hydrogen Sulfide (H_2_S)

#### 3.2.1. Heavy Metal Stress

The application of an H_2_S donor (NaHS) enhanced the activities of the ascorbate–glutathione (AsA–GSH) cycle enzymes and decreased the accumulation of ROS, which further maintained the redox status of the cell and mitigated arsenate (As) toxicity in pea [83] (Table 2). H_2_S maintained Cd and mineral homeostasis in leaves of Cd-stressed foxtail millet [84] and rice [85]. In addition, H_2_S decreased the EC, MDA, and H_2_O_2_ contents, while enhancing photosynthesis in Cd-treated rice seedlings [85]. Meanwhile, under Cd stress conditions, H_2_S reduced oxidative stress, maintained mineral homeostases, upregulated various antioxidant enzymes, and consequently improved the phenotypic appearance of foxtail millet [84]. Significantly, H_2_S not only damages foxtail millet by alleviating Cd but also plays a role in reducing the toxicity of Hg. Correspondingly, NaHS (the H_2_S donor) improved the transcription of bZIP60, a membrane-associated transcription factor, and enhanced the expressions of nonprotein thiols (NPT) and metallothioneins, thereby adequately alleviating Hg toxicity and significantly promoting the growth of rice [4]. Similar results for NaHS, a common donor of H_2_S, were also reported in cauliflower under lead (Pb) stress condition. H_2_S elevated NPT and GSH levels to chelate Pb or clean ROS directly, thus enhancing antioxidant enzyme activities and eventually ameliorating seedling germination and growth [33]. Moreover, H_2_S reduced H_2_O_2_ and MDA contents and upregulated calmodulin gene expression in the leaves of Ni-stressed zucchini seedlings [34] (Table 2). Together, H_2_S may act as an antioxidant to inhibit or clean ROS production to maintain lower MDA and H_2_O_2_ levels and improve mineral homeostasis, thereby preventing the oxidative damage of heavy metals in plants.

#### 3.2.2. Salt Stress

Under salt stress, H_2_S nonselectively regulates the cation channels and overly sensitive salt pathways by maintaining a lower Na^+^ concentration, thus alleviating growth inhibition in wheat seedlings [86] (Table 2). The application of H_2_S could protect cucumber seedlings under salt stress, likely by maintaining Na^+^/K^+^ balance, controlling the endogenous H_2_S levels, and enhancing the antioxidant system under salt stress [87]. Additionally, 0.05 mM NaHS (a donor of H_2_S) was involved in stomatal closure under salt stress, which may function downstream of H_2_O_2_ stomatal movement in the *Vicia faba* [88]. Interestingly, Qi et al. [89] found that H_2_S (100 μM NaHS) played a beneficial role in cucumber seedlings under nitrate stress, and mitogen-activated protein kinase (MAPK)/NO signaling was involved in the process by modulating antioxidant enzyme activities (Table 2). We conclude that plants lower stomatal conductance, and reduce the content of water, Na^+^, and K^+^ in their tissues under salt conditions. Thus, we suggest that H_2_S can reduce Na^+^ concentration, induce stomatal closure, regulate antioxidant enzyme activities, and alleviate the tolerance of plants to salt stress.

#### 3.2.3. Temperature Stress

H_2_S can regulate the gene expression of *VvICE1* and *VvCBF3*, decrease the contents of the superoxide anion radical and MDA, and enhance superoxide dismutase (SOD) activity and the plasma membrane balance of grape leaves challenged by low temperatures [29] (Table 2). H_2_S improved phenylalanine ammonia lyase activity, total phenolic contents, and antioxidant capacity, thereby scavenging ROS accumulation and further improving the tolerance of chilling in banana fruit [90]. Notably, H_2_S (0.5 mM NaHS) maintained higher activities of H^+^-ATPase, cytochrome C oxidase, and succinate dehydrogenase, which consequently enhanced the energy status and improved the chilling tolerance in banana fruit [90]. Meanwhile, Aghdam et al. [37] showed that H_2_S also improved the chilling tolerance of hawthorn fruit by increasing antioxidant enzyme activities and promoting phenol accumulation. Thus, these results indicate that H_2_S can regulate the expression of related genes, improve the activity of antioxidant enzymes, and promote the accumulation of phenolic substances to enhance the tolerance of plants challenged by low temperature stress [91].

With the involvement of Ca^2+^ and calmodulin, an H_2_S donor (NaSH) could improve cell vitality, reduce EC, and enhance accumulation of MDA, consequently ameliorating heat tolerance in tobacco suspension cultured cells [92]. Furthermore, H_2_S reduced S-nitrosoglutathione reductase activity and downstream antioxidant enzyme activities, thereby enhancing tolerance to high temperature stress in poplars [31] (Table 2). The above results highlight a novel signaling mechanism for H_2_S in plant tolerance to high temperatures and suggest a potential strategy to increase tolerance in plants under high temperature stress by genetically modifying H_2_S signaling in plants.

#### 3.2.4. Drought Stress

H_2_S has been reported to reduce the transcriptional expression of ABA receptors in *A. thaliana* under drought stress conditions, thereby enhancing drought tolerance [94] (Table 2). In addition, the exogenous application of an H_2_S donor (NaHS) improved ABA biosynthesis and ABA reactivation gene expression levels in wheat leaves and enhanced the plant and relative water content of wheat seedlings leaves [28]. Meanwhile, H_2_S was found to increase the survival rates of *A. thaliana* seedlings and significantly reduce the stomatal pore size under drought stress [27]. In a further study, the authors found that H_2_S could cause a stomatal closure in *A. thaliana* by regulating the changes in the ion channel activity under drought stress, thus alleviating drought tolerance [2]. Furthermore, Zhang et al. [93] showed that H_2_S (0.1 mM NaHS) modulates antioxidant enzyme activities, effectively increasing chlorophyll content, reducing the MDA content, and increasing the levels of H_2_O_2_ and O_2_⋅^−^, thus increasing drought tolerance in soybean seedlings. Notably, Khan et al. [35] found that NO and H_2_S together clearly enhanced the activities of antioxidant enzymes, thus ameliorating the accumulation of Pro and glycine betaine and protecting wheat seedlings from osmotic stress-induced oxidative stress. Therefore, we conclude that H_2_S, as a gasotransmitter, activates the defense system and maintains the normal functioning of cellular machinery, thereby improving drought resistance in plants.

### 3.3. Nitric Oxide (NO)

#### 3.3.1. Heavy Metal Stress

Pharmacological evidence has suggested that NO increases AhHsp70 expression, decreases AhANT expression, and prevents the mitochondrial permeability transition pore from opening, thus improving mitochondrial physiological properties in peanuts under Al stress conditions [48] (Table 3). We have focused on the participation of NO in plant responses to heavy metal stresses, and its relationship with ROS, as well as its possible role as a cytoprotective signal molecule [95]. In addition, the application of the NO donor S-nitrosoglutathione enhanced the antioxidant defense system, decreased ROS and H_2_O_2_ contents, guaranteed normal indole-3-acetic acid flow towards the roots, and further enhanced Al resistance in wheat seedlings [5,47]. Furthermore, NO enhanced Cd-tolerance in the lichen *Ramalina farinacea* by regulating the balance of ROS and improving changes in mineral nutrient content and metabolites [50]. Thus, NO may regulate the expression of related genes and physiological metabolism, thereby enhancing heavy metal resistance in plants.

#### 3.3.2. Salt Stress

NO not only affects plant growth and development but also enhances the salinity adaptation of plants [105]. Chen et al. [96] reported that NO reduces H_2_O_2_ accumulation and lipid peroxidation and enhances the content of salt stress-reduced GSH and polyphenols, alleviating the oxidative damage in leaves of *Aegiceras corniculatum* as a consequence (Table 3). Meanwhile, application of NO regulated oxidative stress and markedly improved the photosynthetic performance of mustard grown under salt stress [7]. Additionally, Ahmad et al. [97] found that NO improved relative leaf water contents, photosynthetic pigment biosynthesis, osmolyte content, and the antioxidative defense system, thus mitigating the adverse effects on chickpea plants caused by high salt stress. Notably, NO might also downregulate the expression of *PINFORMED* genes, leading to reduced auxin levels, and thus stabilizing *IAA17* for the repressed auxin signaling in *Arabidopsis* [43]. Although NO’s functions in plant salt stressor signalling are now becoming well determined, further research is still needed.

Da Silva et al. [42] reported that NO boosted the enzymatic antioxidant system and improved the nonenzymatic antioxidant GSH under salt stress, thereby regulating the metabolic and physiological changes in tobacco. Evidence suggests that NO enhances the oxidase activity in plants under salt stress [42,98]. The NO donor sodium nitroprusside (100 μM) alleviates salt stress in mustard by improving the growth parameters, photosynthetic traits, and nitrogen metabolism, thereby limiting Na^+^ accumulation and oxidative stress and enhancing Pro content [98]. In addition, NO improved the salt tolerance of *Jatropha curcas* during the establishment of seedlings by inducing an effective antioxidant system, thus inhibiting toxic ions and ROS accumulation, alleviating oxidative stress, and activating the antioxidant defense system [100]. Further, the application of sodium nitroprusside (source of NO) significantly mitigated growth inhibition and enhanced the contents of antioxidants and osmoprotectants in salt-challenged *Pisum sativum* L. [99]. Moreover, the NO donor sodium nitroprusside upregulated antioxidant defense mechanisms and increased ascorbic acid (AsA) contents and total phenolic contents (TPC), thereby effectively increasing the growth and grain yield of wheat under salinity stress [1] (Table 3). Collectively, the accumulating data suggest that NO effectively enhances the growth of plants by upregulating antioxidative defense mechanisms and improving metabolic and physiological changes.

#### 3.3.3. Temperature and Drought Stress

NO (150 µM sodium nitroprusside) improved the quality of wheat grain by increasing the accumulation of gliadin protein and starch and decreasing amylolytic activities under heat stress [101]. However, under low temperature stress, NO (500 μM sodium nitroprusside) lowered EC and MDA accumulation, diminished ROS accumulation, and maintained membrane integrity in cornelian cherry fruits [102] (Table 3). Here, we speculate that NO can regulate plant growth and metabolism by enhancing the activities of antioxidant enzymes and maintaining the integrity of membranes under both heat and low temperature conditions.

Recently, Liao et al. [45] reported that 10 mM NO donor sodium nitroprusside might improve the photosynthetic performance of leaves under drought conditions and alleviate the adverse influence of drought on carbohydrate and nitrogen protection in marigold explants, consequently promoting adventitious rooting. Further, the application of NO significantly improved tricarboxylic acid cycle and antioxidant properties, thus maintaining the redox balance in white clover under water-deficit stress [103]. Meanwhile, NO (0.5 mM sodium nitroprusside) enhanced the antioxidant defense system, upgraded the water status, and decreased oxidative damage and methyl-glyoxal toxicity, thereby increasing drought stress tolerance in wheat seedlings [44]. Under short-term water-deficit stress, the expression of *MtGLR* genes was inhibited by NO, thereby alleviating the loss of water content and embryo axis elongation in *Medicago truncatula* seedlings [104]. Therefore, NO is regarded as a critical moderator of plant growth under drought stress by enhancing the antioxidant defense system, maintaining redox balance, and improving the photosynthetic performance of leaves, thereby alleviating the loss of water content.

### 3.4. Carbon Monoxide (CO)

#### 3.4.1. Heavy Metal Stress

As a signal element, CO (50% CO-saturated aqueous solution) mitigated Cd-induced oxidative damage by regulating GSH and AsA homeostasis in alfalfa roots [57] (Table 4). Similarly, the upregulation of *HO-1* gene expression was related to the depletion of GSH in the roots of alfalfa under Cd stress, leading transiently to an improvement in antioxidative capabilities [106]. Additionally, Meng et al. [107] found that the CO-mediated alleviation of Hg toxicity was closely connected with the accumulation of Pro and the reduction of nonprotein thiols in mustard. Meanwhile, the expression of the *BnHO* gene depressed the generation of O_2_⋅^−^ and H_2_O_2_ and protected *Brassica napus* L. from oxidative injury under Hg stress [3]. Moreover, 1 µM hemin (the water-soluble CO donor) improved the activity of HO-1 transcriptional expression, reduced the accumulation of Zn and the expression of homeostasis-related genes, and strengthened the Zn tolerance of rice seedlings [108]. Thus, under heavy metal stress conditions, CO could improve oxidative stress by enhancing the activities of antioxidative enzymes and antioxidant metabolism in plants.

#### 3.4.2. Salt Stress

The exogenous application of low levels of CO reduced the suppression of seed germination and the damage of seedling leaves in wheat under salt stress by enhancing antioxidant enzyme activities [109] (Table 4). Similarly, CO (1.0 µM hematin), at a low concentration, was able to attenuate the seed germination inhibition under salt stress and counteract the lipid peroxidation in sprouting wheat seeds [110]. Ling et al. [112] found that CO (50% CO aqueous solution) might participate in wheat tolerance against salt stress. Moreover, CO’s moderation of programmed cell death (PCD) and prohibition of root growth were related to the decrease of O_2_⋅^−^ overproduction, partially through the upregulation of SOD and the downregulation of nicotinamide adenine dinucleotide phosphate (NADPH) oxidase expression. Additionally, Zhang et al. [113] reported that 1 μM hematin (an HO-1 inducer and a putative CO donor) increased the levels of cytosolic osmotic substances and antioxidant enzyme activities and reduced the damage to the photosynthetic system under salinity stress, consequently alleviating the growth of seeds and seedlings in *Cassia obtusifolia* L. deriving from salinity stress. A similar result was verified in rice. CO could have a significantly positive influence on attenuating the inhibition of rice seed germination and seedling growth promoted by saline stress and reducing oxidative damage by activating antioxidant enzymes [111]. Moreover, the application of CO improved lipid peroxidation and ureide metabolism, thus protecting the soybean nodule nitrogen fixation and assimilation in soybean plants under salinity stress conditions [58] (Table 4). Therefore, we suggest that CO could regulate antioxidant enzymes, lipid peroxidation, and the photosynthetic system, thereby alleviating salinity stress in plants.

#### 3.4.3. Drought and Temperature Stress

CO brought about significant enhancement in the activities of amylase and antioxidant enzymes, which were beneficial to the mitigation of drought-stress-induced wheat seed germination inhibition and lipid peroxidation [114]. In rice plants, the level of *HO-1* gene expression and HO activity played a significant role in confirming the process of gibberellin-induced PCD in response to drought stress [115] (Table 4). Thus, the level of *HO-1* gene expression, lipid peroxidation, and germination inhibition of plant seeds were improved by CO, thereby enhancing plant tolerance to heavy metals.

Currently, investigations of CO in plant tolerance to temperature stress are scarce. The relative expression of *BnDHNs* in leaves of *Brassica napus* seedlings under low temperature treatment was related to the participation of CO [15] (Table 4).

### 3.5. Methane (CH_4_)

Recently, 0.39 mM CH_4_ (methane-rich water) reduced thiobarbituric acid reactive substances (TBARS) content and enhanced amylase activities and total sugar contents upon Cu stress; in this way, cellular redox homeostasis was re-established in alfalfa seedlings [67] (Table 5). Moreover, CH_4_ partly inhibited Cu-induced Pro production by alternating Pro metabolism [67]. A similar result was confirmed under Al stress; 50% CH_4_ (methane-rich water) alleviated Al toxicity by decreasing Al accumulation in organic-acid-dependent fashion and recovering redox homeostasis [13]. Meanwhile, 1.3 mM CH_4_ pretreatment re-established GSH and redox homeostasis to alleviate Cd toxicity [68]. Further molecular evidence suggested that Al-induced oxidative damage is also alleviated by CH_4_ by regulating antioxidative enzyme activities [13]. Significantly, genetic evidence has demonstrated CH_4_ alleviates Cd accumulation at least partially through the modulation of heavy metal transporters via *miR159* and *miR167* [68]. As an important gaseous molecule, CH_4_ will open a new window into plant resistance to heavy metals and may be applied in phytoremediation processes.

Zhu et al. [65] found that 50% MRW reduced NaCl-induced lipid peroxidation and ROS overaccumulation in alfalfa, and ion homeostasis was re-established. In addition, MRW alleviated the NaCl-induced inhibition of seed germination and oxidative damage, partially by the upregulation of HO-1 [65]. These results could extend our knowledge of CH_4_ in plants and are also crucial to fundamental plant biology.

Under osmotic stress, not only did the sugar content in maize root tissues increase by CH_4_ (exogenously applied 0.65 mM), but the sugar and AsA metabolism in maize seedlings were also regulated by CH_4_ [66]. Further research found a positive role of endogenous NO in CH_4_-enhanced plant tolerance against osmotic stress in mung beans [46]. This research also suggested that NO-regulated redox homeostasis and *S*-nitrosylation might take part in the above CH_4_ action (Table 5). Thus, CH_4_ can be expected to play an advantageous role in plant tolerance against osmotic stress.

## 4. Gasotransmitter Interactions under Adverse Conditions

The crosstalk among gasotransmitters was first discovered in animals. However, in recent years, their interaction has also been confirmed in plants under abiotic stress conditions.

### 4.1. Interaction between H_2_ and NO

**Figure 2:**
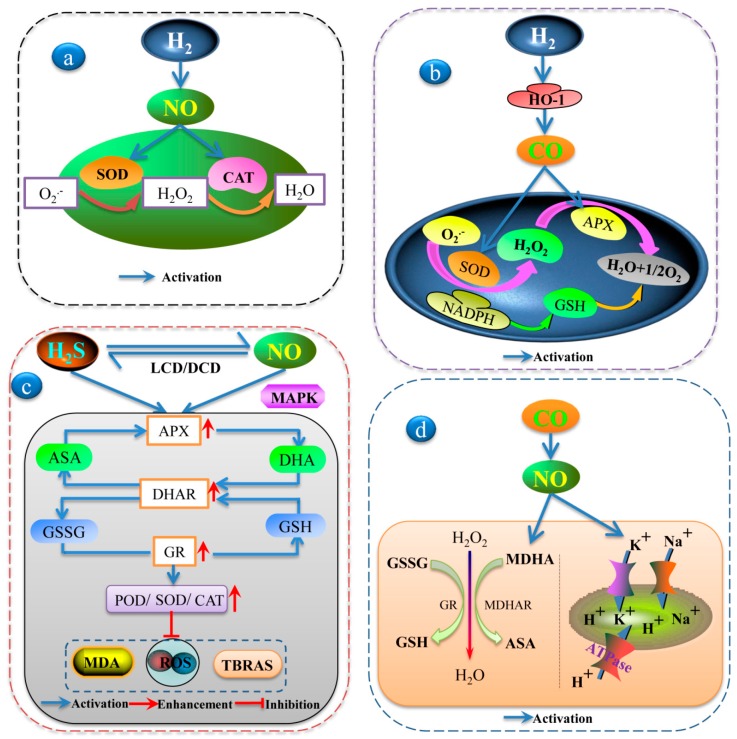
Schematic model of the interaction among H_2_, H_2_S, NO, CO, and CH_4_ in different abiotic stress processes: (**a**) The interaction of H_2_ and NO increased antioxidant defenses in stressed plants. (**b**) Involvement of HO-1 in H_2_ induced different environment stress tolerances in plants. (**c**) The crosstalk between H_2_S and NO upregulated the ASA-GSH cycle, which enhanced the activity of some antioxidant enzymes and alleviated the damage of abiotic stresses to plants. (**d**) CO enhanced abiotic stress tolerance via NO-mediated maintenance of ion balance and the upregulation of antioxidant defense in plants.

Studies on the mechanisms for H_2_ signaling in plants are fragmented, although rapid progress is being made this field. According to recent reports, H_2_ and NO are closely related and alleviate plant tolerance under abiotic stress. NO may play a part downstream in the H_2_ signaling cascade in plants in response to abiotic stressors, such as heavy metal stress [75] and drought stress [116]. Under drought stress conditions, the addition of H_2_ and NO could enhance the antioxidant defense system in stressed plants by reducing ROS production and membrane peroxide and upregulating some antioxidant enzyme activities such as SOD, catalase (CAT), and ascorbate peroxidase (APX) [116] (Figure 2a). In addition, Chen et al. [75] reported that the functional interaction of H_2_ and NO could alleviate Al toxicity symptoms. Our laboratory studies have found that H_2_ and NO are involved in the growth of adventitious roots. H_2_ enhanced NO content by upregulating nitrate reductase activities in cucumber explants [54]. Meanwhile, H_2_ activated the cell cycle and upregulated cell-cycle-related genes and target genes related to adventitious roots via the NO pathway [117]. However, the physiological interaction between NO and H_2_ is more complex in plants. More studies need to be done in the future to clarify this intricate relationship.

### 4.2. Interaction between H_2_ and CO

The importance of the HO-1/CO signalling system in conferring a tolerance of oxidative damage to plants has been well proven. Jin et al. [82] executed a series of physiological and biochemical experiments to indicate the mechanistic depiction of H_2_O_2_ and HO-1 in the H_2_ signalling of alfalfa seedlings exposed to osmotic stress. NADPH oxidase could be the potential source of H_2_-induced H_2_O_2_ generation. The inhibition of NADPH oxidase and the chemical scavenging of H_2_O_2_ could block H_2_-induced HO-1 expression. Additionally, the interaction between H_2_ and CO enhanced SOD, peroxidase (POD), and APX activities, and increased well-known antioxidant GSH contents, thereby improving the antioxidant systems in alfalfa when exposed to paraquat-stressors [23] (Figure 2b). Therefore, it is conceivable that H_2_ can interact with CO as a messenger in plants.

### 4.3. Crosstalk between H_2_S and NO

Along with NO and ROS, H_2_S is involved in numerous stressor responses, including heavy metals, salt, and temperature [26]. H_2_S signal transduction pathways do not always work independently and are closely connected with NO. The two gases share many collaborative downstream signaling pathways and have some similar functions. When it comes to NO downstream of H_2_S, Singh et al. [83] found that H_2_S and NO might both participate in reducing the accumulation of As and triggering the upregulation of the AsA–GSH cycle to counterbalance ROS-mediated damage to macromolecules. Thus, under abiotic stress, NO downstream of H_2_S not only upregulates the AsA–GSH cycle but also alleviates the oxidative damage of plants through signal transduction. MAPK and NO were essential for abiotic stress signaling. The MAPK inhibitor PD98059 and NO scavengers reversed the alleviating effect of H_2_S by enhancing MDA and H_2_O_2_ content and decreasing the antioxidant enzyme activities of SOD, CAT, POD, and APX, as well as the endogenous H_2_S contents and L-cysteine desulfhydrase (LCD) activities under nitrate stress [89]. Obviously, H_2_S primarily affects NO metabolism under environmental stimulations, and H_2_S’s protective role is likely caused by this effect [25] (Figure 2c).

Conversely, when H_2_S was downstream, NO markedly increased the activities of glutathione reductase (GR), APX, POD, SOD, and CAT by enhancing the activities of the H_2_S-synthesizing enzymes LCD and D-cysteine desulfhydrase (DCD), thus alleviating osmotic stress in wheat seedlings [35]. Many redox couples in a cell work together to maintain the redox environment [71], and the GSSG/2GSH couple was one of the principal factors in maintaining cellular redox homeostasis. NO might be located upstream of H_2_S in Bermuda grass’s response to Cd stress by regulating antioxidant enzyme activities (SOD, CAT, POD, and GR) and the nonenzymatic GSH redox state, thus keeping MDA and cell damage at relatively low levels and enhancing Cd tolerance [32] (Figure 2c). NO and H_2_S, which mediate various signaling networks, are crucial elements in the biochemistry and physiology of plants [118]. Together, the synergistic or antagonistic effects of H_2_S and NO might play important roles in the regulation of abiotic stress.

### 4.4. Crosstalk between NO and CO

NO alleviated the harmfulness of ROS, reacted with the CO molecule, and regulated the activation of the antioxidant enzyme system under various stress conditions. Under salinity stress conditions, Xie et al. [119] reported that CO, as well as NO, obviously upregulated the H^+^-pump and the activation of CAT, SOD, APX, GR, and dehydroascorbate reductase (DHAR) or their related transcripts, thereby resulting in the enhancement of the K/Na ratio and the alleviation of ROS in wheat (Figure 2d). In this way, CO could confer an increased tolerance to salt stress by maintaining ion homeostasis and improving the antioxidant system parameters in wheat, both of which were partially mediated by the NO signal. Interestingly, NO also plays a regulatory role in HO/CO systems. HO activity was markedly enhanced by NO and indicated a positive correlation with HO-1 transcript levels. Thus, NO may participate in the UV-B-specific signaling pathway that mediates the HO response under low levels of radiation [120]. We speculate that either NO or CO is located downstream in the form of signal transduction and can play a beneficial role in plant growth under abiotic stressors.

### 4.5. Interaction between CH_4_ and Other Signallings

Maintaining redox homeostasis is a vital mechanism for maintaining plant tolerance against various stressors. NO regulated the ion balance and sugar breakdown in the CH_4_ signaling cascade by reducing ROS production in mung beans under osmotic stress [46]. Notably, HO-1/CO and Ca^2+^ were reported as the downstream signals in CH_4_-induced cucumber adventitious root formation [121]. Both CH_4_ and HO-1 could upregulate the expression of the HO-1 gene, thereby enhancing the total or isozymatic activities of other antioxidant enzymes, including APX, SOD, and POD, and further alleviating the growth inhibition of alfalfa seeds under salt stress [65]. Therefore, we conjecture that CH_4_ may enhance the tolerance of plants via the upregulation of HO-1 under adverse conditions. Additionally, the crosstalk between CH_4_ and other signals in plant tolerance against abiotic stressors remains rare.

## 5. Conclusions and Future Perspectives

Over the years, gasotransmitters, including H_2_, H_2_S, NO, CO, and CH_4_, have become a hot issue in the research of abiotic stress, and there has been major work done in this area. Presently, the available reports suggest that these gasotransmitters are released in plants under different adverse conditions. Importantly, these gasotransmitters enhance plant tolerance to a variety of environmental stimulations, mainly by regulating the activity of antioxidant enzymes, mitigating oxidative stress and lipid pexoxidation, maintaining ion homeostasis, and re-establishing GSH homeostasis. In addition, interactions among gasotransmitters have been confirmed in plants under adverse conditions.

Although a growing body of studies shows that plants may produce gasotransmitters under abiotic stresses, future studies on the biosynthesis of these gasotransmitters should focus on the molecular details of their production pathways in plants. The intricate mechanisms associated with their responses to abiotic stimuli are still a subject of great interest. Therefore, the future study of gasotransmitters in plants should concentrate on their molecular mechanisms and their interactions with each other under abiotic stress. Another question remains: “what are the receptors of these gasotransmitters in plants?” So far, proteomics research has shown that NO has three post-translational modifications to the target protein: metal nitration, tyrosine nitration, and *S*-nitrosation [122]. Additionally, H_2_S may directly modify protein thiol groups [26], and the thiol group in proteins influence cellular function. However, the target proteins of other gasotransmitters remain unclear. Also, these gasotransmitters are indispensable for plant resistance, and more research should be devoted to field experiments with the aim of enhancing agriculture in the areas of yield and quality.

## Figures and Tables

**Table 1:** H_2_ involved in plant abiotic stress tolerance.

Plant Species	Abiotic Stress and Its Effect	H_2_ Roles under Stress	Reference
Alfalfa	Cd stress inhibited root elongation	Improving root growth, re-establishing glutathione homeostasis	[6]
Cabbage	Cd stress reduced the activities of the antioxidant enzyme	Enhancing the activities of the antioxidant enzyme	[8]
Cole	Cd stress affected the balance of glutathione	Governing reduced glutathione homeostasis	[72]
Alfalfa	Cd stress obviously inhibited alfalfa seedling growth	Attenuating damage in alfalfa seedlings, reducing oxidative damage	[73]
Alfalfa	Al stress increased NO production, inhibited root elongation	Improving seedling growth, decreasing NO production	[75]
Maize	Al stress inhibited seed germination, broke the ion balance	Alleviating Al toxicity, decreasing lipid peroxidation	[74]
Rice	Al stress enhanced oxidative damage	Alleviating germination inhibition, re-establishing redox homeostasis	[16]
Alfalfa	Hg stress promoted ROS production	Decreasing ROS production and alleviating oxidative stress	[76]
*Arabidopsis*	Salt stress increased ion outflow	Maintaining ion homeostasis, controlling sodium exclusion	[21]
Rice	Salt inhibited seed germination	The alleviation of oxidative damage	[20]
Cucumber	Temperature stress affected photosynthetic parameters	Altering photosynthetic gas exchange	[78]
Rice	Temperature stress destroyed redox homeostasis	Re-establishing redox homeostasis	[22]
Radish	UV-A stress reduced anthocyanin content	Upregulating the anthocyanin biosynthesis-related genes	[79]
Alfalfa	UV-B stress destroyed the antioxidant defense system	Reducing lipid peroxidation, regulating the antioxidant defence system	[80]
Radish	Short wavelength light stress influenced anthocyanin biosynthesis	Enriching anthocyanin content	[81]
Alfalfa	Oxidative stress enhanced oxidative damage	Increasing levels of the *MsHO-1* transcript, alleviating oxidative stress	[23]
Alfalfa	Drought stress destroyed the redox balance	Modulating stomatal sensitivity, reducing transpirational water loss	[82]
Alfalfa	Drought stress affected the enzyme activity	Elevating H_2_O_2_ levels, the inhibition of NADPH oxidase	[10]

**Table 2:** H_2_S involved in plant abiotic stress tolerance.

Plant Species	Abiotic Stress and Its Effect	H_2_S Roles under Stress	Reference
Rice	Cd stress affected the stability of the membrane	Improving oxidative damage and maintaining ROS homeostasis	[85]
Foxtail millet	Cd stress broke the ion balance	Decreasing electrolytic leakage and enhancing photosynthesis	[84]
Pea	As stress damaged proteins and membranes	Increasing the level of NO, alleviating oxidative damage	[83]
Rice	Hg stress promoted ROS production	Improving the transcription of *bZIP60*, alleviating Hg toxicity	[4]
Cauliflower	Pb stress destroyed GSH levels	Elevating nonprotein thiols and total GSH levels	[33]
Zucchini	Ni stress reduced antioxidant enzyme activity	Enhancing antioxidant enzyme activity and reducing Pro contents	[34]
Wheat	Salt stress inhibited growth of wheat	Decreasing the Na^+^ concentration, alleviating the growth inhibition of wheat	[86]
Cucumber	Salt stress induced oxidative stress	Maintaining Na^+^ and K^+^ homeostasis	[87]
Broad bean	Salt stress affected stomatal sensitivity	Inducing stomatal closure, promoting H_2_O_2_ production	[88]
Cucumber	Salt stress broke the redox balance	Alleviating oxidative damage, upregulating the *CsNMAPK* transcript level	[89]
Grape	Low temperature stress affected the plasma membrane stability	Improving SOD activity and the plasma membrane stability of grape	[29]
Banana	Low temperature disrupted ion stability	Maintaining a higher peel firmness, reducing accumulation of MDA	[90]
Banana	Low temperature stress broke the redox balance	Inhibiting electrolyte leakage and reducing ethylene production	[91]
Hawthorn	Low temperature stress decreased antioxidant enzyme activity	Promoting phenols accumulation and enhancing antioxidant enzyme activity	[37]
Cucumber	Low temperature stress influenced the expression of related genes	Upregulating the expression of Cucurbitacin C synthetase-encoding genes	[30]
Tobacco	Heat temperature stress decreased vitality of cells	Improving vitality of cells and alleviating electrolyte leakage	[92]
Poplar	Heat temperature stress reduced S-nitrosoglutathione reductase activity	Increasing S-nitrosoglutathione reductase activity and reducing reactive oxygen/nitrogen damage	[31]
Soybean	Drought stress affected plant photosynthesis	Enhancing chlorophyll contents and decreasing the production of H_2_O_2_	[93]
*Arabidopsis*	Drought stress changed the expression of drought associated genes	Stimulating the expression of drought associated genes	[27]
*Arabidopsis*	Drought stress influenced the transcriptional expression of the ABA receptor	Decreasing transcriptional expression of ABA receptor	[94]
Wheat	Drought stress changed MDA contents	Increasing antioxidant enzymes activity and reducing MDA contents	[28]
Wheat	Osmotic stress destroyed cysteine homeostasis	Sustaining antioxidant enzymes and cysteine homeostasis	[35]

**Table 3:** NO involved in plant abiotic stresses tolerance.

Plant Species	Abiotic Stress and Its Effect	NO Roles under Stress	Reference
Lichen	Cd stress decreased the content of ionic permeate	Regulating ROS balance, increasing Pro and AsA contents	[50]
Peanut	Al stress promoted the production of harmful substances	Upregulating *AhHsp70* expression and reducing cytochrome c release	[48]
Wheat	Al stress destroyed the antioxidant defense system	Enhancing antioxidant defense, improving H_2_O_2_ levels	[5]
Wheat	Al stress inhibited auxin flow	Improving the oxidized protein levels, guaranteeing normal indole-3-acetic acid flow	[47]
Mangrove	Salt stress induced lipid peroxidation	Reducing hydrogen peroxide content and lipid peroxidation	[96]
*Arabidopsis*	Salt stress broke the ion balance	Downregulating the expression of *PIN* genes, stabilizing *IAA17*	[43]
Tobacco	Salt affected the activity of antioxidant enzymes	Enhancing the activity of antioxidant enzymes and H_2_S levels	[42]
Chickpea	Salt stress increased electrolyte Leakage and the levels of osmolytes	Enhancing the biosyntheses of antioxidant enzymes	[97]
Mustard	Salt stress accelerated oxidative damage	Regulating oxidative stress and photosynthetic performance	[7]
Mustard	Salt stress influenced ion balance	Decreasing electrolytic leakage and K^+^/Na^+^ ratio	[98]
*Pisum sativum* L.	Salt stress triggered the membrane lipid peroxidation	Reducing accumulations of ROS and MDA	[99]
*Jatropha curcas*	Salt stress accelerated toxic ion accumulation	Ameliorating oxidative damage and toxic ion accumulation	[100]
Wheat	Salt stress reduced biomass production and grain yield	Enhancing physiological and biochemical parameters	[1]
Wheat	Temperature stress induced oxidative damage	Enhancing the accumulation of gliadin protein and starch	[101]
Cherry	Temperature stress destroyed membrane integrity	Maintaining antioxidant system activity and membrane integrity	[102]
Marigold	Drought stress induced carbohydrate and nitrogen accumulation	Increasing chlorophyll content and protein content	[45]
Wheat	Osmotic stress created oxidative damage	Enhancing the antioxidant defense system, reducing the methyl-glyoxal content	[44]
White clover	Drought stress influenced metabolic regulation and transform	Inducing changes of metabolic profiles	[103]
Alfalfa	Drought stress inhibited growth physiological processes	Alleviating loss of water content and embryo axis elongation	[104]

**Table 4:** CO involved in plant abiotic stress tolerance.

Plant Species	Abiotic Stress and Its Effect	CO Roles under Stress	Reference
Alfalfa	Cd stress destroyed antioxidation enzymatic activities	Modulating glutathione metabolism	[57]
Alfalfa	Cd induced a loss of plasma membrane integrity, lipid peroxidation	Upregulating expression of *HO-1* gene	[106]
Rapeseed	Hg stress inhibited growth and development	Improving antioxidation capacity and expression of *BnHO-1*	[3]
Mustard	Hg triggered production of O_2_⋅^−^ and H_2_O_2_, as well as peroxides	Improving antioxidative enzymes, reducing oxidative stress	[107]
Rice	Zn stress inhibited root elongation	Downregulating of the expression of homeostasis-related genes	[108]
Wheat	Salt stress induced oxidative damage	Enhancing the activities of antioxidant enzymes	[109]
Wheat	Salt stress caused oxidative damage	Counteracting lipid peroxidation	[110]
Rice	Salt stress inhibited seed germination	Alleviating oxidative damage	[111]
Wheat	Salt stress reduced antioxidant enzyme activities	Decreasing of superoxide anion overproduction	[112]
*Cassia obtusifolia* L.	Salt stress lowered chlorophyll concentration	Alleviating oxidative damage, improving membrane permeability	[113]
Soybean	Salt stress affected the parameters of lipid peroxidation	Improving lipid peroxidation and ureide metabolism	[58]
Wheat	Osmotic stress-induced seed germination inhibition	Increasing in the activities of amylase and antioxidant enzyme	[114]
Rice	Drought stress inhibited HO activity	Improving the level of *HO-1* gene expression and HO activity	[115]
Canola	Temperature stress delayed plant development	Enhancing the expression of *BnDHN* types gene	[15]

**Table 5:** CH_4_ involved in plant abiotic stress tolerance.

Plant Species	Abiotic Stress and Its Effect	CH_4_ Roles under Stress	Reference
Alfalfa	Al stress influenced the physiological roles of alfalfa	Enhancing resistance seedlings, regulating organic acid metabolism	[13]
Alfalfa	Cu-triggered oxidative stress	Increasing amylase activities, reducing Cu accumulation	[67]
Alfalfa	Cd stress decreased the ratio of reduced/oxidized (homo)glutathione	Re-establishing glutathione homeostasis, reducing lipid peroxidation	[68]
Alfalfa	Salt reduced the activities of representative antioxidant enzymes	Reducing reactive oxygen species over accumulation	[65]
Maize	Osmotic stress decreased biomass and relative water contents	Modulating sugar and AsA metabolism	[66]
Mung bean	Osmotic stress broke the ion balance	Re-establishing redox balance, alleviating seed germination inhibition	[46]

## Abbreviations

H_2_	hydrogen gas
H_2_S	hydrogen Sulfide
NO	nitric Oxide
CO	carbon Monoxide
CH_4_	methane
EC	electrolyte leakage
H_2_O_2_	hydrogen peroxide
MDA	malondialdehyde
ROS	reactive oxygen species
ABA	abscisic acid
GSH	glutathione
AsA-GSH	ascorbate–glutathione
AsA	ascorbic acid
HO-1	heme oxygenase-1
HO	heme oxygenase
HRW	hydrogen-rich water
Cu	copper
Cd	cadmium
Al	aluminum
Hg	mercury
Zn	zinc
Ni	nickel
Pb	lead
As	arsenate
UV	ultraviolet radiation
NPT	nonprotein thiols
Pro	proline
pH	potential of hydrogen
SOD	superoxide dismutase
MAPK	mitogen-activated protein kinase
TPC	total phenolic contents
NADPH	nicotinamide adenine dinucleotide phosphate
TBARS	thiobarbituric acid reactive substances
CAT	catalase
APX	ascorbate peroxidase
POD	peroxidase
LCD	L-cysteine desulfhydrase
DCD	D-cysteine desulfhydrase
GR	glutathione reductase
DHAR	dehydroascorbate reductase
PCD	programmed cell death
O_2_⋅^−^	superoxide anion
·OH	hydroxy radicals
